# From Symptoms to Satisfaction: Optimizing Patient-Centered Care in Dry Eye Disease

**DOI:** 10.3390/jcm14010196

**Published:** 2025-01-01

**Authors:** Edoardo Villani, Stefano Barabino, Giuseppe Giannaccare, Antonio Di Zazzo, Pasquale Aragona, Maurizio Rolando

**Affiliations:** 1 Department of Clinical Sciences and Community Health, University of Milan, Eye Clinic San Giuseppe Hospital, IRCCS Multimedica, 20123 Milan, Italy; 2 Ocular Surface & Dry Eye Center, ASST Fatebenefratelli SACCO, University of Milan, 20133 Milan, Italy; stefano.barabino@unimi.it; 3 Eye Clinic, Department of Surgical Sciences, University of Cagliari, 09123 Cagliari, Italy; giuseppe.giannaccare@unica.it; 4 Ophthalmology Operative Complex Unit, University Campus Bio-Medico, 00128 Rome, Italy; antoniodizazzo@gmail.com; 5 Ophthalmology Clinic, Department of Biomedical Sciences, University of Messina, 98100 Messina, Italy; pasquale.aragona@unime.it; 6 Ocular Surface Unit, ISPRE Ophthalmics, 16129 Genoa, Italy; maurizio.rolando@gmail.com

**Keywords:** dry eye disease, ocular surface, tear film, quality of life, symptoms, satisfaction, mental health, therapeutic alliance, depression, PROMs

## Abstract

Dry eye disease (DED) is a multifactorial, chronic, and often relapsing condition with a significant impact on patient quality of life (QoL). Symptoms such as ocular discomfort and visual disturbances are diverse and frequently misaligned with objective clinical signs, complicating diagnosis and management. DED not only interferes with daily activities like reading, driving, and computer use but also imposes a substantial economic burden due to direct healthcare costs and reduced work productivity. Beyond its ocular manifestations, DED has been associated with higher prevalence rates of depression and anxiety, with a complex bidirectional relationship. Patients with DED may experience psychological distress that exacerbates symptoms, leading to a vicious cycle that further impairs QoL. This underscores the importance of integrating mental health screening into the management of DED, particularly for high-risk populations. Optimizing the care of DED patients requires empathy, effective communication, and the establishment of a therapeutic alliance that acknowledges patient experiences and involves them in personalized treatment plans. Such an approach can improve patient satisfaction, enhance treatment adherence, and address both ocular and psychological dimensions of the disease. This paper highlights current evidence on the impact of DED symptoms and its association with mental health and recommends strategies to improve clinical management through a patient-centered approach.

## 1. Introduction

Dry eye (DED) is a common [1], multifactorial [2], symptomatic disease [2], with a potentially relevant, although often underestimated, impact on patient quality of life (QoL) [3,4].

Despite the heterogeneity of epidemiological data, partly due to the use of different DED definitions and patient inclusion criteria, the literature provides strong evidence of the high prevalence of this condition [1], with European data in adults ranging from 18% to 39% [5,6]. 

As discussed in the Tear Film and Ocular Surface (TFOS) Dry Eye Workshop (DEWS) II Definition and Classification Report [2], DED has all the requirements to be recognized as a “disease”, generally defined as “a disorder of structure or function or a condition of illness that results in specific signs or symptoms” [2].

Ocular symptoms, both discomfort and visual disturbance, are a fundamental component of DED [2], which represents the most common reason for seeking medical eye care [1]. DED-related symptoms may include a wide range of patient-reported symptoms [7], and this variety of possible complaints, together with the well-known poor association between signs and symptoms [8], makes DED a potentially frustrating disease for patients and ophthalmologists. 

Moreover, despite our poor knowledge of the natural history and conflicting data on the progressive nature of DED [1], we know that this disease chronically affects millions of people and that a high proportion of them experience symptoms worsening over time, even if treated [9].

DED, insofar as being common, chronic, and symptomatic, may obviously have a relevant impact on patients QoL and even on their mental health, potentially leading to a DED-depression vicious cycle [10,11].

This narrative review summarizes current evidence on dry eye symptoms and their impact on patient QoL, on dry eye patient satisfaction and on the relationship between this disease and mental health.

In the last part of the manuscript, we provide and discuss some recommendations to optimize DED patient management and to improve patient satisfaction, relationship and therapeutic alliance with eye-care providers.

## 2. Literature Search

A PubMed search was conducted using the terms “dry eye disease”, “symptoms”, “quality of life”, “mental health”, and “therapeutic alliance”, combined with Boolean operators (AND/OR). Articles published in English between 2000 and October 2024 were considered. The selection criteria prioritized studies addressing QoL, patient satisfaction, and the psychological impact of DED. We did not conduct a systematic review of the literature but a narrative review focusing on evidence complementary to our clinical experience and necessary to develop recommendations for an optimal approach to managing patients with DED.

## 3. Dry Eye Symptoms

The epidemiological relevance of DED symptoms is well illustrated by population-based cross-sectional studies adopting the Women’s Health Study criteria (severe symptoms of dryness and irritation either constantly or often), which reported DED symptom prevalence between 7% and 24%, with higher values in females, especially after 50 years of age [12,13,14,15,16,17].

The symptoms of DED include ocular surface discomfort and variable blurred vision [18]. 

Ocular discomfort is a hallmark symptom of dry eye disease (DED) and a key component of its definition [7,18]. As highlighted by Tsubota and colleagues, discomfort often manifests before a formal diagnosis of DED and may be crucial for monitoring disease progression and evaluating treatment efficacy [7]. Ocular discomfort encompasses a range of sensations [3,7], including dryness, foreign body sensation, heavy eyelids or eye globe, pain, light sensitivity, discharge, itching, eye fatigue, and redness (some DED patients complain of pink eyes as a cosmetic disfigurement, so redness can also be considered a symptom) [19]. These symptoms reflect the multifaceted nature of ocular discomfort, whose specific nature may vary across DED subtypes, although significant symptom overlap exists. For instance, in aqueous deficiency dry eye (ADDE), symptoms often intensify later in the day, while in chronic meibomian gland dysfunction (MGD) and blepharitis, symptoms often are more pronounced upon awakening, reflecting a diurnal variation [3,7]. From a qualitative point of view, data based on the Asian Dry Eye Society classification suggest that patients with short tear breakup time-type DED may report a heavier sensation, while those with staining-type DED are more likely to experience foreign body sensations [7,20]. Furthermore, photophobia seems to be mainly related to neurosensory abnormalities and might suggest peripheral or central sensitization underlying at least a portion of patient symptoms [21]. Despite these subtype distinctions, symptom overlap is a common feature of DED presentations and there are no definite patterns of symptoms to be used as validated classifier, prognostic, or predictive biomarkers.

On the basis of growing evidence on the potential impact of DED on functional visual acuity [22,23,24], “visual disturbance” was included in the definition of DED proposed by the 2007 TFOS DEWS [18]. Recent studies confirmed that DED is associated with lower reading speed [25] and, even adjusting for age, functional visual acuity is reduced in severe DED [26]. Notably, since standard visual acuity measurements fail to capture the visual impairment associated with DED, clinicians may underestimate its severity [7].

Ocular surface symptoms may be poorly related to objective clinical signs, as ocular surface staining, tear film instability, decreased tear meniscus, or meibomian gland alterations and dropout [2,8]. While there is typically a positive relationship between the severity of symptoms and most objective tests, as well-demonstrated in an interesting paper by Johnson ME [27], the balance of the current data suggests that the association between symptoms and the majority of objective tests is at best moderate, and, thus, the severity of symptoms cannot be estimated with an informative degree of precision from physical signs, or vice versa. This weak correlation, as well as the lack of concordance in their changes over time, was recently confirmed by a sub-analysis of the Dry Eye Assessment and Management (DREAM) study [28]. Furthermore, McMonnies CW [29] recently highlighted a broad range of factors that may contribute to the poor correlation between signs, symptoms, and treatment outcomes. These include mental health comorbidities such as anxiety, depression, hypochondriasis, stress, sleep disturbances, and mood disorders. Such findings emphasize the complex and multifactorial nature of dry eye disease, underscoring the importance of considering both psychological and physical factors in its management.

## 4. Impact of DED Symptoms on Patient QoL

Several studies demonstrated that DED has an adverse effect on overall QoL [1], including aspects of physical, social, and psychological functioning. This disease is able to affect patients’ everyday activities, as driving, reading, using a computer, watching television, work activity, and social life [30,31]. 

Quantitative studies, using utility assessment questionnaires, demonstrated that the impact of DED on QoL might be surprisingly high, showing results similar to those of mild psoriasis and moderate-to-severe angina for mild and severe DED, respectively [32].

The relevance of the impact of DED on patient QoL was confirmed, with a different approach, by an online survey conducted on 706 patients with DED from five European countries, which showed that 31% of patients perceived DED as a “disease” or even a “handicap”, and not as a “discomfort” [33]. 

A sub-analysis of the DREAM Study demonstrated that, in DED patients, greater ocular discomfort was associated with greater interference in several activities of daily living, with a strong correlation between the increasing levels of ocular discomfort and interference with general activity, mood, walking ability, normal work (includes both work outside the home and housework), relationships with others, sleep, and enjoyment of life [34]. Moreover, even in a healthy youthful clinical sample, as recently reported by Asiedu K and colleagues [35], DE symptom severity can have a significant impact on stress scale scores, and an interesting study from Japan showed that the burden of short-break-up-time DED on QOL, as assessed by the Dry Eye-Related Quality-of-Life Score and the Health Utilities Index Mark 3, was as severe as that in ADDE [36]. 

The impact of symptomatic DED extends across multiple dimensions, including vision-related quality of life (VR-QoL), functional limitations, and economic burden. Studies consistently report a significant association between DED and challenges in daily activities such as navigating stairs, recognizing friends, reading road signs, newspapers, and cooking. This association remains statistically significant even after adjusting for factors such as age, gender, and visual acuity [37]. In the domain of VR-QoL, a large population-based cross-sectional study found that DED compromises all VR-QoL domains, with risks comparable to or exceeding those associated with macular degeneration, glaucoma, retinal detachment, and allergic conjunctivitis [38]. Further evidence from a European online survey spanning eight countries (France, Italy, Germany, Greece, the Netherlands, Portugal, Spain, and Sweden) reinforced these findings, showing that participants with self-reported DED exhibited lower functional vision and overall health status than those without DED, with consistent results across regions [39].

In addition to the health-related QoL burden, DED imposes a substantial economic impact on patients and healthcare systems. This burden arises from both direct costs—such as professional healthcare visits, pharmacological and non-pharmacological treatments, and surgical procedures—and indirect costs, including decreased work productivity and lifestyle adjustments. A systematic review quantified these costs in Europe, North America, and Asia, estimating that annual direct costs per patient average $664 in Europe (France, Germany, Italy, Spain, and the United Kingdom), $783 in the United States, and $530 in Japan. Indirect costs, primarily related to productivity loss, range from $741 per patient in Japan to $5362 in the United States [40]. Together, these findings highlight the multidimensional burden of DED, emphasizing its significant impact on functional vision, overall health status, and economic well-being.

## 5. Questionnaires for Assessment of DED Patient Symptoms and QoL

Patient-reported outcome measures (PROMs) are critical tools for the improvement of patient-centered and value-based aspects of healthcare management. 

The previously discussed centrality of symptoms and QoL in DED led to the development and adoption of several questionnaires, generic or disease-specific, each with unique strengths and limitations [41]. 

A recent scoping review [42] identified 49 published PROMs-DED questionnaires, 27 of which had no associated design, validity, and reliability studies. Of the 22 validated questionnaires (17 original and 5 modified), most were designed in English, and the number of items ranged from 1 to 57. Some questionnaires were validated for specific subtypes of disease (Sjögren syndrome, MGD, and Demodex folliculorum) and others for specific population groups (contact lens users or elderly people). 

Here, we briefly discuss an arbitrary selection of the most relevant PROMs-DED questionnaires [3,7,41,43], including The Ocular Surface Disease Index (OSDI), The Impact of Dry Eye on Everyday Living (IDEEL), the Dry Eye-Related Quality-of-Life Score (DEQS), the Dry Eye Questionnaire-5 (DEQ-5), and the Symptoms Assessment in Dry Eye (SANDE) (Table 1).

Among DED-specific PROMs, the OSDI [44] is the most extensively utilized tool in clinical trials and research due to its strong validation and widespread adoption. It comprises 12 items designed to evaluate the frequency of symptoms, their impact on vision-related activities, and environmental triggers. Despite its advantages, the OSDI has limitations, including a narrower scope that does not fully capture the psychological and social dimensions of DED. Furthermore, its scores can be influenced by ocular comorbidities, particularly in older populations, potentially complicating the interpretation of results in heterogeneous patient groups.

In contrast, the IDEEL [45] provides a broader and more comprehensive assessment of QoL, encompassing symptoms, daily activities, and treatment satisfaction. Its extensive 57-item structure allows for an in-depth evaluation of DED’s multifaceted impact on patients’ lives. However, this level of detail comes at the cost of practicality, as the time required for completion may limit its feasibility in routine clinical practice. This makes the IDEEL more suitable for research settings where comprehensive data collection is prioritized.

For settings where efficiency is essential, shorter PROMs, such as the DEQ-5 [46] and SANDE [47], are particularly valuable. The DEQ-5, derived from the longer DEQ, focuses on the severity of symptoms with high sensitivity, making it a practical choice for screening. The SANDE, on the other hand, employs a visual analog scale to assess symptom frequency and severity, providing a quick and easy-to-administer tool for monitoring. While both instruments prioritize brevity and efficiency, they lack the depth needed for a full assessment of QoL and the psychosocial impact of DED, limiting their utility for comprehensive evaluations.

Cultural and demographic factors further influence the choice of PROMs. For example, the DEQS [48], a 15-item questionnaire focusing on bothersome symptoms and their impact on daily life, has demonstrated strong psychometric properties. However, its validation is primarily limited to Japanese populations, raising concerns about its generalizability across diverse cultural settings. Similarly, generic QoL instruments such as the NEI VFQ-25 [49] have been adapted for use in DED studies, offering valuable insights into visual function across domains such as ocular pain, dependency, and social impacts. While these tools provide a broader perspective, they lack the specificity required for detailed evaluations of DED-specific symptoms and QoL, highlighting a trade-off between general applicability and disease-focused precision.

Despite the availability of numerous tools, no single questionnaire fully encapsulates the multifactorial nature of DED or aligns seamlessly with standardized diagnostic criteria. This observation underscores the complexity of evaluating DED and highlights the importance of selecting PROMs tailored to specific disease subtypes, conditions, or population groups for which they have been validated. To ensure the reliability and applicability of selected questionnaires, it is crucial to assess not only their validation status but also the methodological rigor of their design and their usability in different clinical and research contexts. Equally important is the need to establish clear guidelines on how, in which contexts, for which patient populations, and with what purpose these tools should be applied. This approach would help clinicians and researchers maximize the utility of existing PROMs while addressing specific clinical and investigational needs. Future efforts should prioritize the development of globally validated and culturally adaptable questionnaires that integrate symptoms, visual function, and quality-of-life measures. Combined with clearer strategies for their implementation, such tools could bridge existing gaps by enhancing diagnostic precision, supporting regulatory requirements, and facilitating comparability across studies, ultimately improving the quality of care for patients with DED.

## 6. DED Patient Satisfaction

Patient satisfaction is a critical component in the patient-centered management of DED, as it directly reflects the effectiveness of treatment strategies and influences the patient–physician relationship. Understanding satisfaction levels and their determinants is essential for optimizing care and improving outcomes.

An insightful study by Schaumberg et al. highlighted the prevalence of dissatisfaction among DED patients, reporting that approximately 20% of patients were somewhat or extremely dissatisfied with their treatment [50]. This finding underscores the need to better address patient expectations and outcomes in DED management.

A cross-sectional study conducted across 10 USA optometry and ophthalmology practices offered a closer look at patient reliance on over-the-counter (OTC) treatments. Among 158 symptomatic DED patients, nearly 75% reported using OTC medications, yet only 64.2% expressed satisfaction with these treatments, and just 37.3% experienced meaningful symptom relief [51]. This gap between widespread usage and limited efficacy points to the need for more effective therapeutic options and patient education regarding available interventions.

In a more comprehensive investigation at the Cornea Service of the Massachusetts Eye and Ear Infirmary (Boston, MA, USA), satisfaction was assessed across multiple dimensions using a detailed questionnaire. The study, involving 91 DED patients, evaluated their understanding of the condition, perceived ease of following medical advice, treatment effectiveness, satisfaction with eye care, and overall outlook on the disease [52]. Over 90% of participants reported satisfaction with their understanding of DED and believed their treatments were beneficial. Furthermore, 76% found it easy to follow their doctors’ recommendations, while 48% expressed optimism about the long-term prospects of their condition. However, as this study was conducted at a high-profile referral center specializing in DED, these results may overestimate satisfaction levels and may not fully reflect the experiences of the general patient population.

A particularly intriguing aspect of patient satisfaction, with significant implications for the patient–physician relationship, is the alignment—or lack thereof—between patient and clinician assessments of DED severity. Studies from the USA [53] and Taiwan [54] reveal that clinicians often underestimate the severity of DED as perceived by patients. These discrepancies, occurring in 41% to 51% of cases, highlight the importance of integrating patient-reported outcomes into clinical evaluations to achieve a more holistic understanding of the disease’s impact.

Taken together, these findings underscore the complexity of managing DED and the critical role of patient satisfaction in optimizing therapeutic strategies. Addressing both the subjective experiences of patients and potential mismatches in clinician–patient assessments is essential for improving outcomes and advancing patient-centered care.

## 7. DED and Mental Health

Several studies have demonstrated a strong association between DED and mental health conditions, particularly depression and anxiety. These findings were summarized in a meta-analysis [55], reporting that the prevalence of these conditions in DED patients is nearly three times higher than in controls, regardless of DED etiology or patient ethnicity. This underscores the systemic impact of DED beyond ocular symptoms.

The Beijing Eye Study [56], a large population-based analysis, found a significant correlation between the frequency of dry eye symptoms and self-rated depression scores, even after adjusting for key demographic and lifestyle factors. Interestingly, no significant associations were observed for other major ocular diseases, such as glaucoma or cataracts, emphasizing the unique role of DED in mental health.

A recent meta-analysis by Basilious et al. [57] further explored this relationship, reporting a 40% prevalence of depression and anxiety in DED patients, with significantly higher odds compared to controls (OR: 1.81 for depression; OR: 2.32 for anxiety). While depression and anxiety severity correlated strongly with DED symptoms, no significant relationships were found with objective signs [57]. Additionally, studies suggest that patients with pre-existing depression exhibit poorer tear film stability and higher rates of undiagnosed DED, highlighting a potentially bidirectional relationship [58,59].

A landmark study leveraging data from the NIH’s All of Us Research Program [60] revealed significantly higher rates of depressive disorders, anxiety, bipolar disorder, and schizophrenia in DED patients compared to controls, with the strongest associations observed in Black participants. This research, notable for its diverse cohort and robust adjustments for confounders, emphasized the need for routine mental health screening in DED management, particularly in underserved populations and suggested that DED may independently contribute to mental health issues.

As discussed by Weatherby et al. [61], DED and mental health conditions appear to interact synergistically, worsening patients’ overall quality of life. However, longitudinal studies provide conflicting data on whether mental health improvements directly influence DED outcomes [62,63]. These findings highlight the importance of integrating psychological care into DED treatment strategies to effectively address this complex interplay.

## 8. Discussion on Management

Empathy, dialogue, and communication aimed to involve the patient in a real therapeutic alliance are elements of crucial importance in the management of DED patients. Evidence from literature suggests that the psychoanalytic concept of therapeutic alliance, referring to the sense of collaboration, warmth, and support between the client and therapist, could be applied to different fields of medicine, especially to chronic pain and chronic symptomatic diseases [64,65]. The therapeutic alliance construct is based on 3 main components: the therapist–patient agreement on goals, the therapist–patient agreement on interventions, and the bond between patient and therapist.

**Figure 1:**
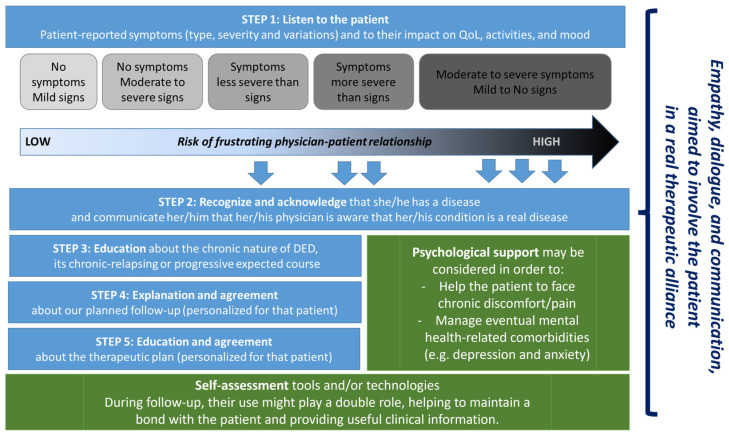
Conceptual framework: therapeutic alliance with DED patients.

As previously discussed, DED patients may have chronic moderate-to-severe symptoms, significantly affecting their QoL, even in cases with mild objective signs [8]. Moreover, they may have previous experience of several ineffective and frustrating medical examinations and therapies [50,51]. This underscores the importance of adopting a patient-centered approach (Figure 1) that not only addresses the physical symptoms of DED but also acknowledges its psychological and social dimensions [28,29]. 

The first step, in order to build a good relationship with these patients, is to recognize and acknowledge that they have a disease and to communicate to them that their physician is aware that their condition is a real disease. Recognizing the weak correlation between DED signs and symptoms, as confirmed by the DREAM study [28], clinicians must rely on patient-reported outcomes to guide diagnosis and treatment, ensuring the patient feels understood and supported.

The second relevant step is to listen to these patients, dedicating time and attention to patient-reported symptoms and to their impact on QoL, activities, and mood. Besides being an important element to build the relationship, this patient-centered approach can provide useful clinical information, including symptom severity, type, pattern, and changes over time. Studies, such as those by McMonnies CW [29], emphasize the necessity of considering comorbidities like depression, anxiety, and stress, which are frequently associated with DED and exacerbate its impact on QoL.

The following steps should be aimed to build a patient–physician partnership, involving collaborative care and education. Education should provide information about the chronic nature of DED, its chronic-relapsing or progressive expected course, and the treatment plan. It might be important to explain to the patient how follow-up visits will be scheduled and that, over the course of the treatment path, the therapies will be dynamic and individually tailored. Ophthalmologists should adopt this personalized approach even for non-pharmacological treatments, which should be carefully chosen, explained, and prescribed, instead of generically and hastily recommended. Moreover, we should keep in mind and reassure the patient about one of our most relevant goals: the decrease of symptom severity, intrusiveness, and impact on QoL.

During follow-up, the use of self-assessment tools and/or technologies might play a double role, helping to maintain a bond with the patient and providing useful clinical information. As highlighted in this review, validated PROMs such as the OSDI and DEQ-5 can offer valuable insights into symptom severity and QoL, facilitating adjustments to the treatment plan [44,46]. Furthermore, proper integration of these tools into routine clinical practice can support a more dynamic and responsive approach to care.

Finally, given the already mentioned association and possible vicious cycle between DED and depression and anxiety, measures aimed at improving mental health status could greatly benefit certain DED patients. Unfortunately, both animal models and clinical studies suggest that the usage of selective serotonin reuptake inhibitors and serotonin noradrenaline reuptake inhibitors affects the ocular surface, potentially worsening DED signs and symptoms [66,67]. Considering this, a multidisciplinary approach that integrates the expertise of ophthalmologists, psychologists, and other healthcare professionals may be essential to address the complex and multifaceted nature of DED. This strategy would enable the simultaneous management of ocular symptoms, psychological comorbidities, and the broader impact of the disease on patient quality of life, as emphasized throughout this review. However, the implementation of such an approach poses challenges, including resistance from healthcare providers unfamiliar with multidisciplinary frameworks, coordination difficulties among specialists, and variable acceptance of psychological or collaborative care by patients. Overcoming these barriers requires targeted education for both clinicians and patients, highlighting the benefits of integrated care models. Additionally, psychological treatments, such as cognitive behavioral therapy, could support DED patients by addressing chronic discomfort, visual symptoms, and mental health-related comorbidities like depression and anxiety, ultimately improving their quality of life. This paper underscores the need for an integrative management strategy that combines personalized treatment, psychological support, and effective communication to optimize outcomes for patients with DED.

## Figures and Tables

**Table 1:** Descriptive Analysis of a selection of Patient-Reported Outcome Dry Eye Questionnaires. QoL: Quality of Life; DED: Dry Eye Disease.

Name (Acronym)	Contents (N of Items)	Type of Response	Score Interpretation	Advantages	Disadvantages
Ocular Surface Disease Index (OSDI)	Symptoms, Visual Function, Environment Triggers (12 items)	5-point Likert scale (0–4): 0 = none of the time, 4 = all of the time	Range: 0–100. Severity: 0–12 = normal, 13–22 = mild, 23–32 = moderate, 33–100 = severe	Widely used, validated, evaluates symptoms and impact on vision-related activities	Limited focus on emotional aspects; influenced by ocular comorbidities in elderly patients
Impact of Dry Eye on Everyday Living (IDEEL)	Symptoms, Daily Activities, Treatment Satisfaction (57 items)	Multi-dimensional questionnaire	Scores calculated for each domain separately	Comprehensive; covers symptoms, QoL, and treatment satisfaction	Complex; time-consuming for routine clinical use
Dry Eye-Related Quality-of-Life Score (DEQS)	Daily Life Impact, Bothersome Symptoms (15 items)	4-point scale for symptoms (1–4) and 0–4 scale for bothersome aspects	Range: 0–100. Higher scores indicate worse QoL	Focuses on QoL; strong psychometric properties	Primarily validated in Japanese populations; limited cross-cultural adaptation
Dry Eye Questionnaire-5 (DEQ-5)	Symptom Severity (5 items)	Frequency and intensity ratings for dryness and discomfort	Range: 0–22. Higher scores indicate greater severity	Short, easy to administer, high sensitivity for symptom screening	Lacks detail on QoL and symptom complexity
Symptoms Assessment in Dry Eye (SANDE)	Symptom Frequency and Severity (2 items)	Visual Analog Scale for symptom severity and frequency	Range: 0–100 for each item	Simple, quick, suitable for monitoring and screening	Lacks detail on QoL or specific symptom domains
National Eye Institute Visual Function Questionnaire (NEI VFQ-25)	Visual Function Across Multiple Domains (25 items)	5-point Likert scale for most items	Range: 0–100 for each subscale. Higher scores indicate better visual function	Broad assessment of vision-related quality of life	Not specific for DED; may miss key symptoms unique to DED
